# Deep Learning vs. Radiomics for Predicting Axillary Lymph Node Metastasis of Breast Cancer Using Ultrasound Images: Don't Forget the Peritumoral Region

**DOI:** 10.3389/fonc.2020.00053

**Published:** 2020-01-31

**Authors:** Qiuchang Sun, Xiaona Lin, Yuanshen Zhao, Ling Li, Kai Yan, Dong Liang, Desheng Sun, Zhi-Cheng Li

**Affiliations:** ^1^Institute of Biomedical and Health Engineering, Shenzhen Institutes of Advanced Technology, Chinese Academy of Sciences, Shenzhen, China; ^2^Department of Ultrasonic Imaging, Peking University Shenzhen Hospital, Shenzhen, China; ^3^Ultimage Lab, Suzhou, China; ^4^Peng Cheng Laboratory, Shenzhen, China

**Keywords:** breast cancer, deep learning, radiomics, axillary lymph node metastasis, breast ultrasound, peritumoral region

## Abstract

**Objective:** Axillary lymph node (ALN) metastasis status is important in guiding treatment in breast cancer. The aims were to assess how deep convolutional neural network (CNN) performed compared with radiomics analysis in predicting ALN metastasis using breast ultrasound, and to investigate the value of both intratumoral and peritumoral regions in ALN metastasis prediction.

**Methods:** We retrospectively enrolled 479 breast cancer patients with 2,395 breast ultrasound images. Based on the intratumoral, peritumoral, and combined intra- and peritumoral regions, three CNNs were built using DenseNet, and three radiomics models were built using random forest, respectively. By combining the molecular subtype, another three CNNs and three radiomics models were built. All models were built on training cohort (343 patients 1,715 images) and evaluated on testing cohort (136 patients 680 images) with ROC analysis. Another prospective cohort of 16 patients was enrolled to further test the models.

**Results:** AUCs of image-only CNNs in both training/testing cohorts were 0.957/0.912 for combined region, 0.944/0.775 for peritumoral region, and 0.937/0.748 for intratumoral region, which were numerically higher than their corresponding radiomics models with AUCs of 0.940/0.886, 0.920/0.724, and 0.913/0.693. The overall performance of image-molecular CNNs in terms of AUCs on training/testing cohorts slightly increased to 0.962/0.933, 0.951/0.813, and 0.931/0.794, respectively. AUCs of both CNNs and radiomics models built on combined region were significantly better than those on either intratumoral or peritumoral region on the testing cohort (*p* < 0.05). In the prospective study, the CNN model built on combined region achieved the highest AUC of 0.95 among all image-only models.

**Conclusions:** CNNs showed numerically better overall performance compared with radiomics models in predicting ALN metastasis in breast cancer. For both CNNs and radiomics models, combining intratumoral, and peritumoral regions achieved significantly better performance.

## Introduction

Breast cancer is the leading malignancy in females (1). Axillary lymph node (ALN) metastasis status is one of the most important factors in guiding treatment decision making in breast cancer (2). Traditionally, the nodal status was assessed by surgical methods such as sentinel lymph node biopsy (SLNB) and axillary lymph node dissection (ALND) (3). According to the guideline from American Society of Clinical Oncology, SLNB is considered to have a high overall accuracy ranging from 93 to 97.6% with a relatively low false negative rate (FNR) ranging from 4.6 to 16.7% in detecting axillary metastasis (4). However, these surgical approaches have been considered controversial due to the invasiveness, potential complications, and possible overtreatment (3–6).

Ultrasound is a widely-used tool in breast cancer assessment as it is non-invasive, radiation-free, real-time and well-tolerated in women. Previous studies have shown that axillary ultrasound (AUS) may provide useful information relevant to ALN status in breast cancer (7). However, AUS alone has moderate sensitivity and may not be a reliable predictor for nodal metastasis (7, 8). Recently, imaging-based machine learning approaches have been demonstrated promising in cancer diagnosis. There are two most popular machine learning approaches: radiomics analysis and convolutional neural networks (CNN). Radiomics analysis relies on a pipeline including extraction of numerous handcrafted imaging features, followed by feature selection and machine learning-based classification. Handcrafted radiomics features extracted from the breast tumor area have been demonstrated predictive in ALN metastasis, with FNRs ranging from 13.9 to 25% (9, 10). However, handcrafted features are limited to the current knowledge of medical imaging, which may limit the potential of the predictive model. Deep learning improves this handcrafted pipeline by automatically learning discriminative features directly from images. Recent studies have shown that deep CNN-based approaches can achieve state-of-the-art performance in lesion detection and cancer diagnosis (11–13). To our knowledge, no studies have assessed breast ultrasound-based CNN in predicting ALN status for breast tumor.

Most studies have focused on mining predictive imaging features within the tumor, while the surrounding tissues were ignored. Previous evidence has shown that the peritumoral region—the tumor-adjacent parenchyma immediately surrounding the tumor mass—may offer valuable outcome-associated information (14–16). Two recent studies have demonstrated that handcrafted imaging features from peritumoral region in Dynamic Contrast-Enhanced MRI (DCE-MRI) are associated with sentinel lymph node metastasis (9) and pathological complete response to neoadjuvant chemotherapy (17) in breast cancer. Here, we hypothesize that deep CNN built based on intra- and peritumoral regions in breast ultrasound could provide relevant information in predicting ALN status. We are interested in comparing the performance of deep CNNs and radiomics models. Additionally, breast cancer can be classified into different molecular subtypes with distinct prognosis and respond differently to specific therapies (18). Therefore, we further assessed if deep CNNs or radiomics models combining imaging features and molecular subtypes could offer improved accuracy.

In this hypothesis-driven study, we first developed deep CNNs and radiomics models based on intratumoral, peritumoral, and combined regions in breast ultrasound images for predicting ALN metastasis. We then aimed to find out how on each region deep CNNs performed compared with radiomics models.

## Materials and Methods

### Study Population

The study was approved by the Ethics Committee of Peking University Shenzhen Hospital (PUSH). Informed consent was waved from all patients by the ethics committee of PUSH. From the pathology and radiology databases in PUSH, a retrospective search was performed to recruit female patients with breast cancer between January 2016 and December 2018. The inclusion criteria were patients (1) with histologically-confirmed primary breast cancer, (2) with pretreatment breast ultrasound images, (3) with known ALN metastasis status determined by final histopathology, (4) with known molecular subtypes, and (5) without neoadjuvant chemotherapy prior to SLNB or ALND. The exclusion rules were that patients (1) with very small region of interest in the ultrasound images (<100 pixels) and (2) without SLNB or ALND. Finally, 479 patients with 479 breast tumors (136 positive and 343 negative ALNs) were included in this study. This cohort was randomly divided into a training cohort of 359 patients and a testing cohort of 120 patients at a ratio 3:1. The patient recruitment pathway was shown in Figure S1.

The baseline clinical and histopathological data were derived from patient medical records, including age, histological grade, immunohistochemistry (IHC) results and ALN status (positive or negative). According to the 2017 St Gallen International Expert Consensus, each patient was classified into one of four molecular subtypes: human epidermal growth factor receptor-2 (HER2) positive, triple-negative, Luminal A, and Luminal B (18). The status of HER2, ER, progesterone receptors (PR) and Ki-67 was assessed by IHC. Based on the IHC results, the subtype can be determined.

### Ultrasound Image Acquisition

The breast ultrasound examinations were performed by breast radiologists in our center using the Hitachi Ascendus ultrasound system equipped with 13–3 MHz linear array transducers. The examinations and assessments were conducted according to the 5th edition of Breast Imaging Reporting and Data System (BI-RADS) presented by American College of Radiology (ACR) (19). The parameters were set as follows: depth, 4–5 cm; brightness gain, 10–25 dB; dynamic range, 70 dB; frame rate, 26 frame *per second*. Patients were placed in supine or lateral position. The field of view was set to have the pectoralis muscle at the deepest aspect of the image. The focal zone was adjusted to be centered at the lesion. Ultrasound images were acquired and documented into the Picture Archiving and Communication Systems (PACS). For each lesion, five images were selected from PACS by a breast radiologist (XL with 5 years' experience in breast radiology) and used in our study according to the following scheme: (1) an image along the longest axis of lesion. (2) an image orthogonal to the first image. (3) three images at other angles where the lesion was clearly presented. The five images together represented the ultrasonographic features of a 3D lesion from different angels. For all 479 patients, we finally obtained 2395 images in total, including 1715 images (343 patients) in the training cohort and 680 images (136 patients) in the testing cohort.

### ROI Delineation

**Figure 1:**
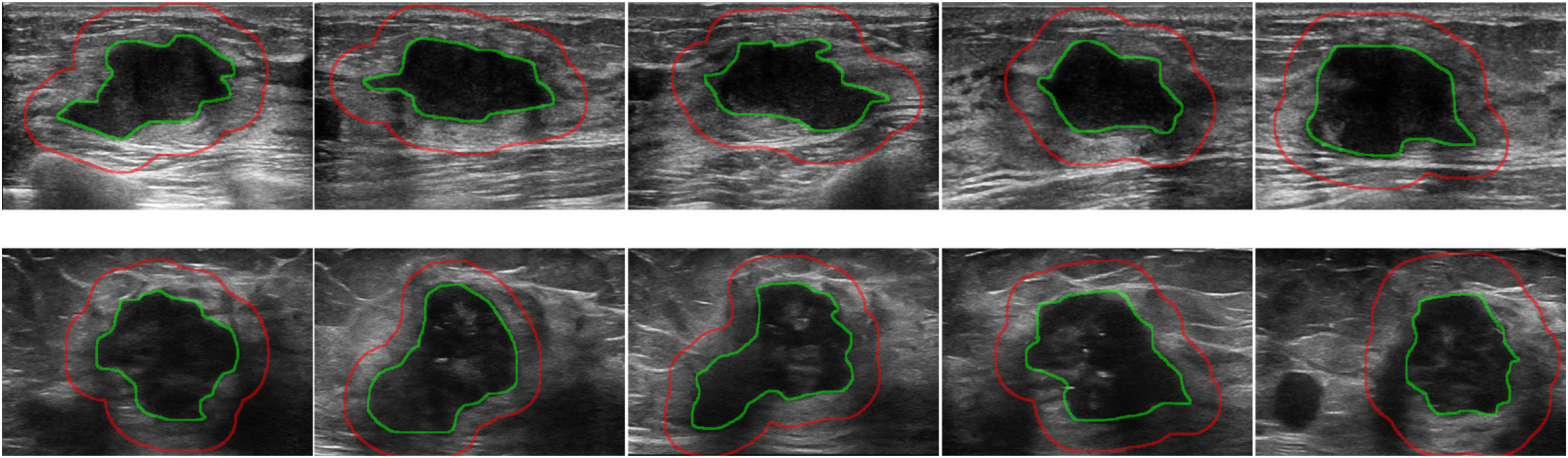
Examples of ultrasound slices overlapped with intratumoral regions (green) and peritumoral regions (red) from two patients. **(Top)** A patient with positive ALN. **(Bottom)** A patient with negative ALN.

The tumor region in each ultrasound image was manually delineated using the ITK-SNAP software (http://www.itksnap.org) by one radiologist (XL) who were blinded to the clinical and histopathological data of patients. A second breast radiologist (DS with 12 years' experience in breast radiology) reviewed all the delineations. Any disagreement between the two raters was resolved by discussion and consensus. The peritumoral regions were obtained by dilating the delineated tumor contour by ~5 mm based on a standard morphological dilation operation using an inhouse program implemented in Matlab 2016b (MathWorks, Natick, MA). For each ultrasound slice, three region of interest (ROI) images were finally obtained: the intratumor ROI, the peritumor ROI, and the combined ROI that merged the intratumor and the peritumoral regions. Examples of ultrasound slices overlapped with intratumoral and peritumoral ROIs for two patients were shown in Figure 1.

### Deep Learning With DenseNet

**Figure 2:**
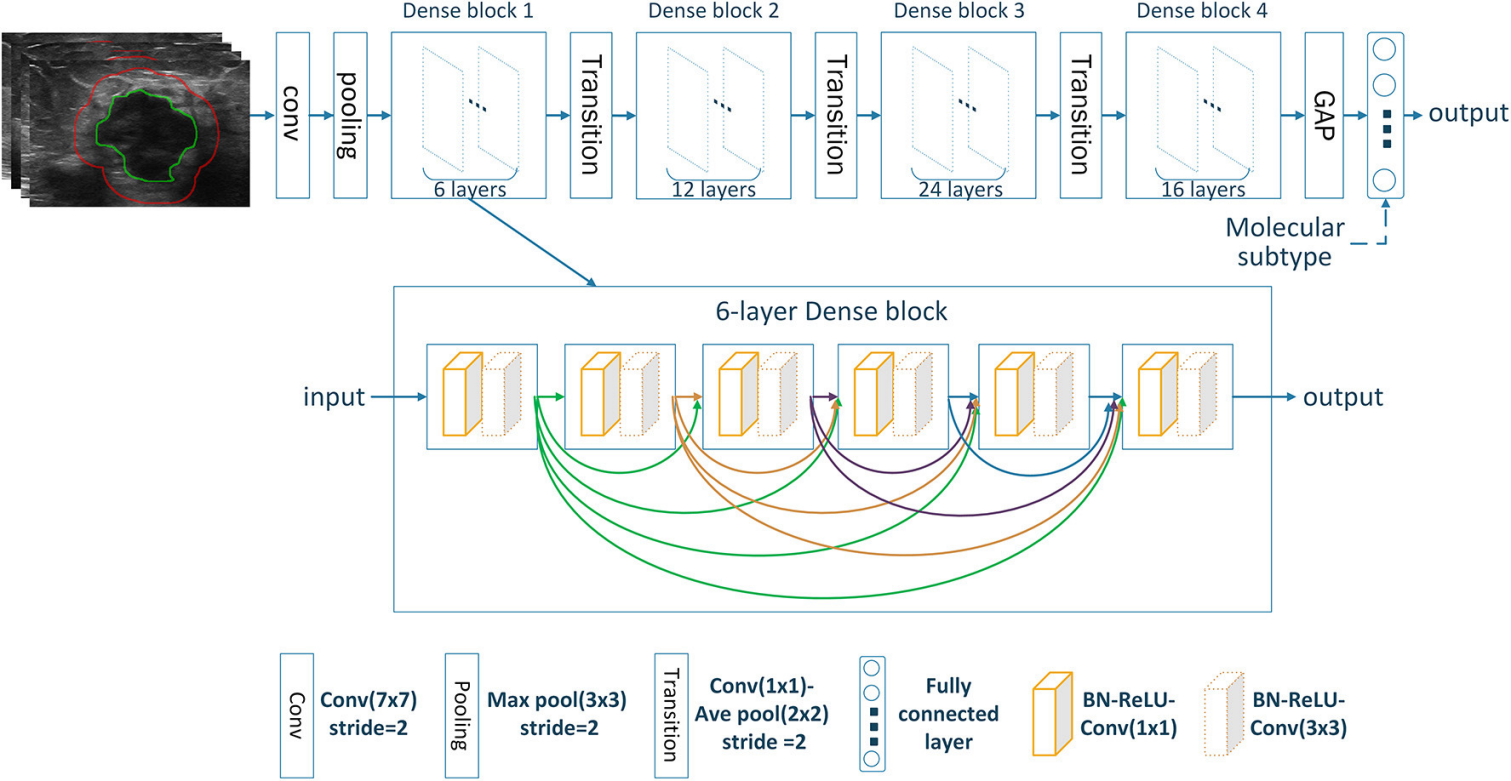
The architecture of the deep CNN used in our study.

Deep CNN can automatically learn discriminative features from imaging data by stacking multiple convolutional layers. Among different CNN variants, densely connected convolutional network (DenseNet) has shown superior classification performance as it strengthens feature propagation while reduces parameter number (20). This is accomplished by connecting each layer to every other layer in a feed-forward fashion with less computational complexity. Here, our model was built based on the standard DenseNet-121 (20). All ROI images were resized into 224 × 224. The resized ROI images were used as input and transformed through the chained convolutional layers, yielding a class probability vector as the prediction results. The network was trained from scratch with cross entropy loss function and Adam optimizer with a learning rate of 0.0001, a batch size of 16, and a regularization weight of 0.0001. In the training cohort, data augmentation approaches including random rotation, random shear and random zoom were employed before the training procedure to avoid possible overfitting. The network was implemented on Keras (https://keras.io/) with the TensorFlow library as the backend (https://www.tensorflow.org/). The architecture of the image-only CNN network was shown in Figure 2. The details of the convolutional network implementation can be found in Table S1.

### Deep Learning-Based Predictive Model Building

For predicting the nodal status, three image-only CNN models, including the intratumoral CNN, the peritumoral CNN and the combined-region CNN, were built with the DenseNet based on the intratumor ROI images, the peritumor ROI images, and the combined ROI images, respectively. Furthermore, three corresponding image-molecular models were also built based on the DenseNet by using both ROI images and molecular subtype information as the network input. Specifically, the molecular subtype information was incorporated as input to the fully-connected layers of the DenseNet, as shown in Figure 2.

### Radiomics Feature Extraction and Selection

For each ultrasound slice, 104 radiomics features were extracted from each of the three ROI areas by using an open-source toolbox named Pyradiomics (https://pyradiomics.readthedocs.io) (21). Three groups of features were extracted, including shape features, intensity features, and texture features, as summarized in Table S2. Eleven shape features describing the geometric characteristics of the ROI were extracted. Eighteen intensity features describing the first-order distribution of the ROI intensities were extracted. Seventy-five texture features were computed to describe the patterns, or the high-order distributions of the ROI intensities with five different methods, including the gray-level co-occurrence matrix (GLCM), gray-level run length matrix (GLRLM), gray level size zone matrix (GLSZM), gray level dependence matrix (GLDM), and neighborhood gray-tone difference matrix (NGTDM). The detailed definitions of the radiomics features used can be found in two articles (22, 23). Having high-dimensional radiomics features, feature selection is required to reduce the dimension and avoid overfitting. Here an efficient machine learning-based wrapper algorithm, Boruta, was used to select a subset of features that were relevant to the prediction outcome (24). Boruta evaluated feature relevance iteratively by comparing the importance of original features with that achieved by artificially added random features, yielding an all-relevant subset of features that was considered optimal for the classification task. Here we used the R package Boruta for Boruta feature selection.

### Radiomics-Based Predictive Model Building

Based on the selected radiomics features, three image-only radiomics models were built using random forest algorithm (25) based on the intratumor ROI, the peritumor ROI, and the combined ROI, respectively. Correspondingly, three image-molecular radiomics models were also built using random forest by integrating ROI images and molecular subtype information as the input. After testing different settings, the tree number of all random forest classifiers was set to 300. Gini index was used as importance measure (26). The R package randomForest was used for random forest classification.

### Statistical Analysis

The difference in age, histological grades and molecular subtypes between training and testing cohorts was assessed with χ^2^ test or Wilcoxon rank-sum test, where appropriate. All 12 prediction models (3 image-only CNNs, 3 image-only radiomics models, 3 image-molecular CNNs and 3 image-molecular radiomics models) were trained on the training cohort and evaluated on the testing cohort. Because each tumor had five ultrasound images, there were five corresponding prediction outcomes in the form of class probabilities. Among them, the median probability was chosen as the final prediction of each tumor and was used for statistical analysis. The prediction performance was assessed by the area under the receiver operating characteristic (ROC) curve (AUC), accuracy (ACC), sensitivity (SEN), specificity (SPE), positive predictive value (PPV), and negative predictive value (NPV). The AUCs between two models were statistically compared using a DeLong test (27). All statistical analyses were performed with R software, version 3.5.1 (https://www.r-project.org/). All statistical tests were two sided, and *p* < 0.05 indicated significant.

## Results

Patient and tumor characteristics are summarized in Table 1. No significant difference was found in patient age, histological grades, molecular subtypes and ALN status between the training and testing cohorts (*p* = 0.457 to 0.844).

**Table 1:** A summary of patient and tumor characteristics of the study population.

**Variables**	**All retrospective** **patients (*n* = 479)**	**Training** **cohort (*n* = 359)**	**Testing** **cohort (*n* = 120)**	***P*-value**	**Prospective** **cohort (*n* = 16)**	***P*-value**
Age (mean ± SD)	48.7 ± 11.1	48.9 ± 10.9	47.9 ± 11.9	0.844	49.8 ± 11.3	0.680
Histological grade				0.755		0.556
I	187 (39.0%)	140 (39.0%)	47 (39.2%)		8 (50.0%)	
II	249 (52.0%)	190 (52.9%)	59 (49.2%)		7 (43.7%)	
III	43 (9.0%)	29 (8.1%)	14 (11.6%)		1 (6.3%)	
Molecular subtype				0.457	-	-
Luminal A	45 (9.4%)	33 (9.2%)	12 (10.0%)		-	
Luminal B	322 (67.2%)	239 (66.6%)	83 (69.2%)		-	
HER2 positive	57 (11.9%)	44 (12.3%)	13 (10.8%)		-	
Triple negative	55 (11.5%)	43 (11.9%)	12 (10.0%)		-	
ALN				0.829		0.418
Positive	136 (28.4%)	101 (28.1%)	35 (29.2%)		6 (37.5%)	
Negative	343 (71.6%)	258 (71.9%)	85 (70.8%)		10 (62.5%)	

*P-values were calculated by using χ^2^ test or Wilcoxon rank-sum test. P-values in the fifth column were calculated between training and testing cohorts. P-values in the seventh column were calculated between training and prospective cohorts*.

### Image-Only Deep CNNs vs. Radiomics Models

**Figure 3:**
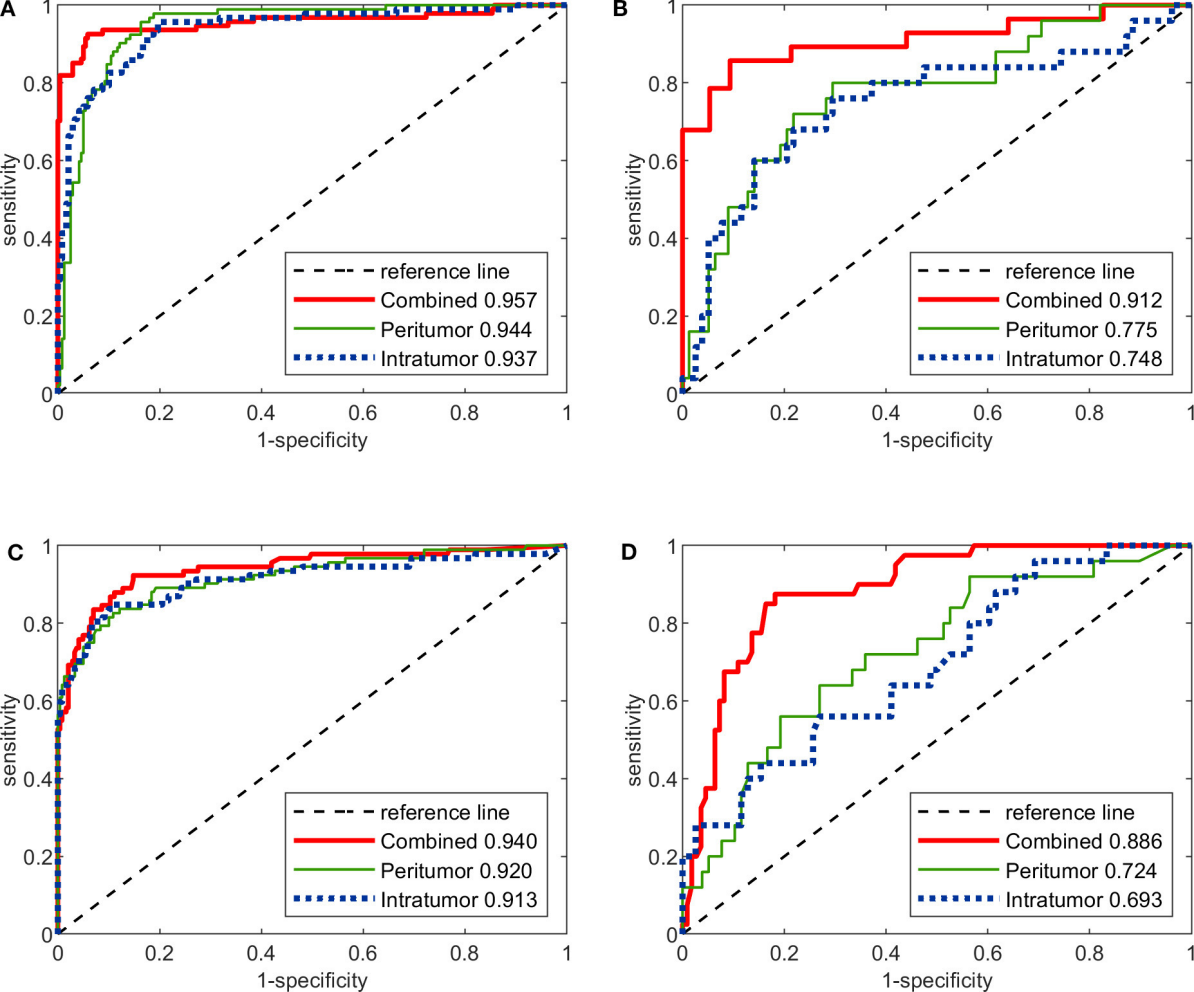
The ROC curves of the three image-only deep CNNs and the three image-only radiomics models in both training and testing cohorts. **(A)** ROC curves of image-only CNNs in training cohort. **(B)** ROC curves of image-only CNNs in testing cohort. **(C)** ROC curves of image-only radiomics models in training cohort. **(D)** ROC curves of image-only radiomic models in testing cohort.

The predictive performance of the three image-only deep CNNs and the three image-only radiomics models in both training and testing cohorts is summarized in Table 2. Their ROC curves in both training and testing cohorts are shown in Figure 3, respectively. The radiomics feature selection results can be found in Table S3. Among all six image-only models, the combined-region CNN achieved the best performance with a highest AUC of 0.912 and a highest accuracy of 89.3% in the testing cohort. In the testing cohort, the CNN built on each region performed better than the corresponding radiomics model built on the same region in terms of AUC and accuracy, but the differences of AUCs between the CNNs and their corresponding radiomics models were not statistically significant (Image-only CNN vs. Radiomics: Intratumoral: AUC 0.748 vs. 0.693, *p* = 0.534; Peritumoral: AUC 0.775 vs. 0.724, *p* = 0.531; Combined-region: AUC 0.912 vs. 0.886, *p* = 0.601).

**Table 2:** A performance summary of the image−only CNNs and image−only radiomics models in training and testing cohorts in predicting ALN metastasis of breast cancer.

**Model**		**Dataset**	**AUC**	**ACC (%)**	**SEN (%)**	**SPE (%)**	**PPV (%)**	**NPV (%)**
Image−only CNN	Intra	Training	0.937 (0.908, 0.968)	84.6 −	95.7 (80.4, 98.9)	80.3 (76.6, 95.4)	65.2 (60.7, 87.8)	98.0 (92.5, 99.5)
		Testing	0.748 (0.622, 0.874)	71.8	76.0 (48.0, 92.0)	70.5 (60.3, 94.9)	45.2 (39.2, 77.8)	90.2 (84.0, 96.7)
	Peri	Training	0.944 (0.920, 0.969)	87.0 −	95.7 (89.1, 100.0)	83.7 (78.7, 91.6)	69.3 (63.5, 81.1)	98.0 (95.3, 100.0)
		Testing	0.775 (0.665, 0.886)	72.8 −	80.0 (56.0, 92.0)	70.5 (64.1, 92.3)	46.5 (40.9, 72.7)	91.7 (85.3, 96.8)
	Cmb	Training	0.957 (0.926, 0.989)	93.7 −	92.6 (86.2, 97.9)	94.1 (90.8, 99.6)	86.1 (80.0, 98.8)	97.0 (94.5, 99.1)
		Testing	0.912 (0.834, 99.0)	89.3 −	85.7 (67.9, 96.4)	90.7 (84.0, 100.0)	77.4 (66.7, 100.0)	94.4 (89.0, 98.6)
Image−only radiomics	Intra	Training	0.913 (0.870, 0.956)	87.9 −	84.8 (75.0, 91.3)	89.1 (86.6, 95.8)	75.0 (70.9, 88.6)	93.8 (90.6, 96.4)
		Testing	0.693 (0.573, 0.812)	68.9 −	56.0 (32.0, 100.0)	73.1 (28.2, 100.0)	40.0 (30.0, 100.0)	83.8 (81.0, 100.0)
	Peri	Training	0.920 (0.882, 0.958)	87.3 −	82.6 (72.8, 93.5)	89.1 (80.3, 96.7)	74.5 (63.6, 89.9)	93.0 (90.2, 97.0)
		Testing	0.724 (0.609, 0.839)	70.9 −	64.0 (48.0, 100.0)	73.1 (38.5, 91.0)	43.2 (32.4, 66.7)	86.4 (83.5, 100.0)
	Cmb	Training	0.940 (0.908, 0.973)	87.1 −	92.3 (81.3, 96.7)	85.2 (82.3, 95.9)	70.0 (66.1, 88.1)	96.7 (92.8, 98.6)
		Testing	0.886 (0.831, 0.942)	83.3 −	87.5 (72.5, 97.5)	81.8 (75.5, 91.8)	63.6 (55.7, 78.7)	94.7 (90.0, 98.9)

*ACC, AUC, SEN, SPE, PPT, and NPV are short for accuracy, area under the receiver operating characteristic curve, sensitivity, specificity, positive prediction value, and negative prediction value, respectively. Intra, Peri and Cmb indicate the intratumoral model, the peritumoral model and the combined−region model, respectively. Statistical quantifications were demonstrated with 95% confidential interval (CI), when applicable*.

### Image-Molecular Deep CNNs vs. Radiomics Models

**Figure 4:**
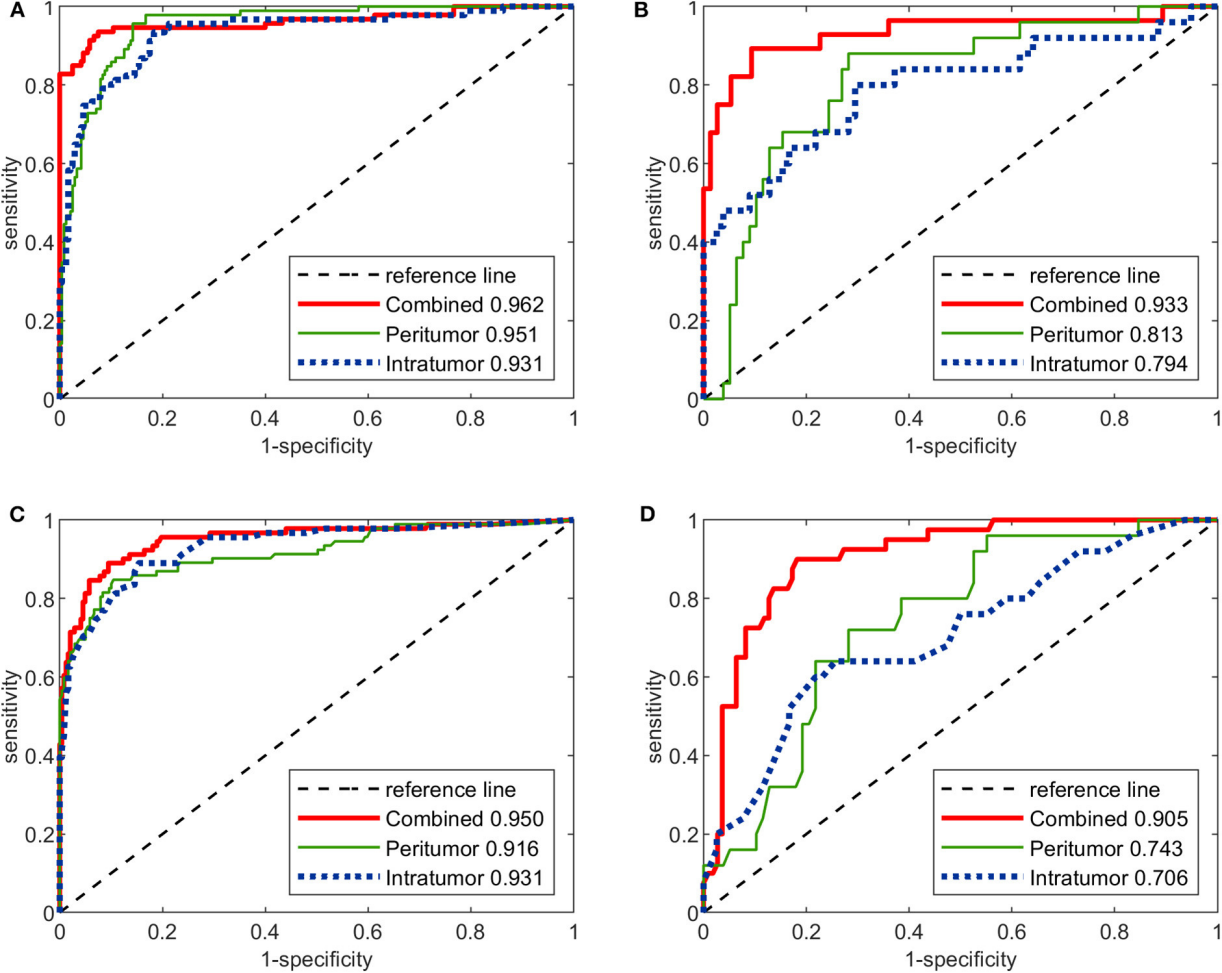
The ROC curves of the three image-molecular deep CNNs and the three image-molecular radiomics models in both training and testing cohorts. **(A)** ROC curves of image-molecular CNNs in training cohort. **(B)** ROC curves of image-molecular CNNs in testing cohort. **(C)** ROC curves of image-molecular radiomics models in training cohort. **(D)** ROC curves of image-molecular radiomic models in testing cohort.

The performance of the three image-molecular CNNs and the three image-molecular radiomics models is summarized in Table 3. Their ROC curves in both training and testing cohorts are shown in Figure 4. From Tables 2, 3, it can be found that the overall performance of the image-molecular models was slightly higher than those of their corresponding image-only models in the testing cohort, but no significant AUC differences were found between them. Among all 12 predictive models built in our study, the image-molecular CNN model built based on the combined-region achieved the best performance with a highest AUC of 0.933, a highest accuracy of 90.3% and a highest NPV of 0.958 in the testing cohort. All image-molecular CNNs achieved higher AUCs and higher accuracy than their corresponding radiomics models built based on the same tumoral region, but there were no significant differences between their AUCs (Image-molecular CNN vs. Radiomics: Intratumoral: AUC 0.794 vs. 0.706, *p* = 0.308; Peritumoral: AUC 0.813 vs. 0.743, *p* = 0.334; Combined-region: AUC 0.933 vs. 0.905, *p* = 0.531).

**Table 3:** A performance summary of the image−molecular CNNs and image−molecular radiomics models in training and testing cohorts in predicting ALN metastasis of breast cancer.

**Model**		**Dataset**	**AUC**	**ACC (%)**	**SEN (%)**	**SPE (%)**	**PPV (%)**	**NPV (%)**
Image−molecular CNN	Intra	Training	0.931 (0.898, 0.964)	84.9 −	93.4 (78.2, 98.9)	81.7 (76.3, 96.3)	65.9 (60.3, 88.8)	97.0 (92.0, 99.5)
		Testing	0.794 (0.677, 0.911)	72.8 −	80.0 (48.0, 96.0)	70.5 (60.3, 100.0)	46.5 (40.3, 100.0)	91.7 (84.5, 98.0)
	Peri	Training	0.951 (0.928, 0.973)	88.5 −	95.7 (90.2, 100.0)	85.8 (79.9, 91.6)	72.1 (65.2, 81.1)	98.1 (96.1, 100.0)
		Testing	0.813 (0.717, 0.909)	75.7 −	88.0 (64.0, 100.0)	71.8 (64.1, 92.3)	50.0 (43.1, 74.1)	94.9 (88.2, 100.0)
	Cmb	Training	0.962 (0.933, 0.990)	92.8 −	93.5 (83.9, 97.8)	92.5 (89.6, 100.0)	82.9 (77.9, 100.0)	97.4 (94.1, 99.1)
		Testing	0.933 (0.864, 1.000)	90.3 −	89.3 (75.0, 100.0)	90.7 (82.7, 100.0)	78.1 (67.5, 100.0)	95.8 (91.0, 100.0)
Image−molecular radiomics	Intra	Training	0.931 (0.898, 0.964)	85.8 −	89.0 (78.0, 95.6)	84.6 (78.7, 94.6)	68.6 (61.5,84.8)	95.3 (91.7, 98.1)
		Testing	0.706 (0.583, 0.828)	71.8 −	64.0 (40.0, 88.0)	74.4 (46.1, 89.7)	44.4 (32.9, 66.7)	86.6 (81.1, 93.9)
	Peri	Training	0.916 (0.877, 0.955)	88.2 −	84.8 (76.1, 91.3)	89.5 (85.8, 95.4)	75.7 (70.0, 87.2)	93.9 (90.9, 96.5)
		Testing	0.743 (0.640, 0.847)	71.8 −	72.0 (60.0, 100.0)	71.8 (39.7, 85.9)	45.0 (33.8, 60.7)	88.9 (86.1, 100.0)
	Cmb	Training	0.950 (0.921, 0.980)	90.1 −	89.0 (81.3, 96.7)	90.5 (81.5, 96.7)	77.9 (65.9, 90.4)	95.7 (93.1, 98.7)
		Testing	0.905 (0.855, 0.956)	84.0 −	90.0 (77.5, 97.5)	81.8 (74.5, 93.6)	64.3 (56.5, 82.9)	95.7 (91.2, 98.9)

*ACC, AUC, SEN, SPE, PPT, and NPV are short for accuracy, area under the receiver operating characteristic curve, sensitivity, specificity, positive prediction value, and negative prediction value, respectively. Intra, Peri and Cmb indicate the intratumoral model, the peritumoral model and the combined−region model, respectively. Statistical quantifications were demonstrated with 95% confidential interval (CI), when applicable*.

### Assessment of Peritumoral and Intratumoral Regions

The predictive value of different tumoral regions were assessed by comparing the models built with the same machine learning methods (CNN or radiomics). It was observed that for the image-only CNNs and image-only radiomics models, the AUCs of the peritumoral models were slightly higher than those of the intratumoral models in the testing cohort, and their AUC differences were not significant (Image-only Peritumoral vs. Intratumoral: CNN: AUC 0.775 vs. 0.748, *p* = 0.746; Radiomics: AUC 0.724 vs. 0.693, *p* = 0.707). Similar results have been observed for the image-molecular models (Image-molecular Peritumoral vs. Intratumoral: CNN: AUC 0.813 vs. 0.794, *p* = 0.806; Radiomics: AUC 0.743 vs. 0.706, *p* = 0.647).

The image-only CNNs and image-only radiomics models built based on combined-region achieved higher AUCs than their corresponding models built based on either the intratumoral or peritumoral region in the testing cohort, where the AUC differences between them were significant (Image-only Combined-region vs. [Peritumoral, Intratumoral]: CNN: AUC 0.912 vs. [0.775, 0.748], [*p* = 0.049, *p* = 0.031]; Radiomics: AUC 0.886 vs. [0.724, 0.693], [*p* = 0.014, *p* = 0.004]). The image-molecular CNNs and image-molecular radiomics models built based on combined-region also achieved higher AUCs. For image-molecular models, the difference between AUCs of the combined-region CNN and either the intratumoral CNN or peritumoral CNN was significant (Image-molecular Combined-region vs. [Peritumoral, Intratumoral]: CNN: AUC 0.933 vs. [0.813, 0.794], [*p* = 0.048, *p* = 0.046]; Radiomics: AUC 0.905 vs. [0.743, 0.706], [*p* = 0.006, *p* = 0.003]).

### Prospective Validation

To further validate the CNNs and radiomics models, we performed a validation study using a relatively small prospective cohort. From November 18 2019 to December 12 2019, 16 breast cancer patients (6 node positive and 10 node negative) with 80 breast ultrasound images (each had 5 images as described in section Ultrasound Image Acquisition) were finally enrolled for analysis. Age, grade, and node status were obtained for the 16 patients and were summarized in Table 1. All six image-only prediction models were tested. As we did not obtain IHC results, the image-molecular models were not tested. The model performance in this prospective cohort was summarized in Table 4. The ROC curves of all tested models were shown in Figure S2. We observed that the CNN built on the combined region achieved the highest AUC of 0.95 and the highest accuracy of 81.3%, where two patients with positive node and one patient with negative node were misclassified. In general, CNNs outperformed radiomics models; prediction models built on combined region outperformed those built on either intratumor region or peritumor region only. The results were consistent with previous observation on the retrospective cohort.

**Table 4:** A performance summary of the image-only CNNs and image-only radiomics models in the prospective cohorts in predicting ALN metastasis of breast cancer.

**Model**		**AUC**	**ACC (%)**	**SEN (%)**	**SPE (%)**	**PPV (%)**	**NPV (%)**
Image-only CNN	Intra	0.767	75.6	50.0	90.0	75.0	75.0
	Peri	0.850	75.0	50.0	90.0	75.0	75.0
	Cmb	0.950	81.3	66.7	90.0	80.0	81.8
Image-only radiomics	Intra	0.533	68.8	33.3	90.0	66.7	69.2
	Peri	0.533	68.8	33.3	90.0	66.7	69.2
	Cmb	0.833	81.3	83.3	80.0	71.4	88.9

*ACC, AUC, SEN, SPE, PPT, and NPV are short for accuracy, area under the receiver operating characteristic curve, sensitivity, specificity, positive prediction value, and negative prediction value, respectively. Intra, Peri and Cmb indicate the intratumoral model, the peritumoral model and the combined-region model, respectively*.

## Discussion

The major findings of this study were that deep CNN, built by combining intratumoral and peritumoral regions in breast ultrasound images, outperformed radiomics models in predicting ALN metastasis. Although imaging-based machine learning approaches have been demonstrated useful in assessing breast cancers, few studies have been done on evaluating the value of intra- and peritumoral regions in metastasis prediction (9), and no studies have investigated how breast ultrasound-based deep CNNs performed compared with radiomics models. In this study, we first developed three types of CNN models based on intratumoral, peritumoral, and combined regions, respectively in ultrasound images for assessing the nodal metastasis, and further compared the performance of the three CNNs with three radiomics models built based on the same regions in nodal metastasis prediction. Moreover, we evaluated if further benefit can be obtained by integrating ultrasound images and molecular subtype information into the predictive models. Note that besides a high AUC, a high NPV is also important as accurately identifying patients with negative nodes [~65% in all breast cancer patients (28)] helps to avoid axillary overtreatment and reduce associated serious complications.

Identification of possible association between breast ultrasound features and ALN status has undoubtful clinical benefit. In clinical routine, the axilla can be staged clinically by palpation or surgically by SLNB or ALND. Although SLNB has less severe complications compared with ALND, it is not risk-free and SLNB- associated complications have been reported in large prospective trials (6). As palpation is inaccurate (29), AUS is performed to provide more relevant information. AUS alone has a reported sensitivity of 39–60%, specificity of 90–96%, PPV of 80–91%, and NPV of 75–83% (6, 30, 31). This implied that despite of an acceptable specificity above 90%, prior to surgery about 40–60% of nodal metastases cannot be found by AUS and about 20–25% of patients with a negative AUS have been assessed as modal metastases after surgery. In case of suspicious ALN, AUS alone or combined with ultrasound-guided needle biopsy is performed for axillary staging to select patients who would benefit from ALND. A recent meta-analysis has shown that the use of AUS in stratifying patients directly to fast-track ALND without SLNB leads to overtreatment in up to two-thirds of patients (32). These data indicated that AUS alone is not sufficiently accurate for axillary staging.

Recent studies have shown the value of radiomics features from primary lesion in predicting the lymph node metastasis for different cancer sites, e.g., CT radiomics features in colorectal cancer (33), MRI/CT radiomics features in bladder cancer (34, 35) and esophageal cancer (36). For breast cancer, two recent studies have assessed the value of radiomics features extracted from the primary tumor region at DCE-MRI and diffusion-weighted MRI (DWI) in predicting sentinel lymph node metastasis, where the reported AUC, sensitivity and specificity ranging from 0.805 to 0.869, 0.700–0.778, and 0.747–861 respectively (9, 10). In our study, we built three image-only radiomics models by using both peri- and intratumoral regions in multiple ultrasound slices per lesion. The combined-region radiomics model achieved an AUC of 0.886, a sensitivity of 87.5% and a specificity of 81.8% on the testing cohort, which were comparable with the previous radiomics models built with MRI.

Although promising, an efficient radiomics analysis heavily relies on a handcrafted image processing pipeline comprising three tightly coupled steps: feature extraction, feature selection and machine learning model building. Small variations in each stage may affect the prediction accuracy and stability (37). Deep CNN improves this pipeline by automatically learning predictive features on its own and yields a class probability vector as output. Currently, CNN-based learning methods have achieved diagnostic accuracy levels in skin cancer (11) and retinal diseases (12, 13), which have been unattainable via radiomics approaches. For breast cancer, a comparative study (38) demonstrated that CNN was superior to radiomic analysis in terms of a significantly higher AUC (0.88 vs. 0.81, *p* < 0.001) for classification of enhancing lesions as benign or malignant at MRI. Another comparative study in Kooi et al. (39) also demonstrated that CNN was superior to radiomics-based software in detection of mammographic breast lesions. In our study, all six CNNs (three image-only and three image-molecular) achieved higher AUC and accuracy than corresponding radiomics models built on the same regions on both training and testing cohorts. Note that in our results the differences between their AUCs (CNN vs. radiomics) were not significant (DeLong *p* > 0.05).

Most image analysis studies on breast cancer was focused on the intratumoral region. Evidences have demonstrated that imaging features of peritumoral regions can offer outcome-related information. Several studies have demonstrated that the enhancement patterns of tumor-adjacent parenchyma in DCE-MRI were associated with chemotherapy response (14), local recurrence (15), and survival (16) in breast cancer. In a recent study (40) the grade of peritumoral edema identified in breast MRI has been independently associated with disease recurrence. In study by Zhou et al. (41), it was demonstrated that the peritumoral stiffness assessed by ultrasound elastography of malignant breast lesions was higher than that of benign lesions. A 2017 study (17) was the first attempt to extract radiomics features from both intratumoral and peritumoral regions in breast DCE-MRI, where the features successfully predicted the pathological complete response to neoadjuvant chemotherapy. A more recent 2019 study (9) for the first time demonstrated the feasibility of predicting sentinel lymph node metastasis by using intratumoral and peritumoral radiomics features in DCE-MRI, achieving an AUC of 0.806 and an NPV of 82.4% with radiomics features only. Our study has shown the value of peritumoral ultrasonographic CNN features in predicting nodal metastasis with an AUC of 0.775 and an NPV of 91.6%. By combining both intra- and peritumoral regions, the CNN achieved a significantly better AUC of 0.912 and an NPV of 94.4%. The FNRs of the image-only CNN model built by combining the intra- and peritumoral regions achieved 5.9, 9.3, and 10% in the training, testing, and prospective data sets, respectively, which were superior to the image-only radiomics model with FNRs of 14.8, 18.25, and 20% in the training, testing, and prospective data sets, respectively. The FNRs of the CNN model were comparable with those of SLNB [4.6 to 16.7% (4)] and were higher than the radiomics models [13.9 to 25% (9, 10)] reported previously. By integrating the molecular subtype information, all the obtained image-molecular models, either CNN or radiomics, achieved slighter higher AUCs and NPVs.

The biological mechanism underlying the peritumoral imaging features and their association with clinical outcomes remains unclear. Many cancer studies have shown that biological changes in the tissue immediately surrounding the breast tumor mass might be potential predictive or prognostic markers, such as peritumor lymphovascular invasion (42, 43), peritumoral lymphocytic infiltration (44), and peritumoral edema (45). In study by Zhao et al. (46) it was suggested that vascular endothelial growth factor (VEGF)-C/D induced peritumoral lymphangiogenesis may be one mechanism that leads to metastatic spread. In study by Wu et al. (16) the prognostic peritumoral features were associated with the tumor necrosis factor (TNF) signaling pathway that has been involved in oncogenic angiogenesis, invasion, and metastasis (47). Further studies are warranted to determine how the underlying biological changes were reflected by peritumor imaging features.

Our study has several limitations. The first limitation was the limited population size which may lead to bias. Larger patient population from more centers should be involved in future to improve the machined learning-based models. The population size of the prospective cohort is particularly small, where significant bias may occur. We will recruit more prospective data in future to further evaluate our methods in clinical practice. The second limitation was that all image data was obtained on the same type of ultrasound machine. In future we will evaluate our models on more heterogeneous image data acquired with different machines. Moreover, we built our CNNs and radiomics models using only ultrasound images and molecular subtypes. In future we will build more comprehensive models by incorporating more clinical and pathological data. Our future research also includes the exploring of biological mechanism underlying the association between intratumoral/peritumoral imaging features and nodal metastasis. We will also assess the possible incremental value of the tumoral ultrasonographic features over the AUS in axillary staging.

In conclusion, CNNs built on tumoral regions in ultrasound images allowed accurate prediction of ALN metastasis, which achieved higher AUC and NPV than radiomics models. Either CNNs or radiomic models built on peritumor regions performed slighter better than those built on intratumor regions, while combining both intra- and peritumoral regions achieved significantly better AUCs and higher NPVs. Further integrating the molecular subtype information into either CNNs or radiomics models can slightly benefit the performance.

## Data Availability Statement

To achieve repeatability, the data set of this study, including pretrained CNN models, imaging data of the prospective cohort, statistical analysis, and the Python implementation, was deposited into the Mendeley data library (https://data.mendeley.com/datasets/rc32mg38rb/draft?a=2333e5fd-e7b1-4603-b06e-b609d79bab11).

## Ethics Statement

The studies involving human participants were reviewed and approved by Ethics Committee of Peking University Shenzhen Hospital. The ethics committee waived the requirement of written informed consent for participation.

## Author Contributions

DS, Z-CL, and DL conceived and designed the study. XL collected the clinical and image data and performed image pre-processing. QS, YZ, LL, and KY analyzed the image data and performed the statistical analysis. QS wrote the manuscript. All authors approved the final manuscript.

### Conflict of Interest

LL was employed by the company Ultimage Lab. The remaining authors declare that the research was conducted in the absence of any commercial or financial relationships that could be construed as a potential conflict of interest.
