# Supplementary material for: Deep Learning vs. Radiomics for Predicting Axillary Lymph Node Metastasis of Breast Cancer Using Ultrasound Images: Don't Forget the Peritumoral Region
Source: Front Oncol. 2020 Jan 31;10:53. doi: 10.3389/fonc.2020.00053 (PMC7006026; doi:10.3389/fonc.2020.00053)
Supplement: Supplementary file 1 [file Data_Sheet_1.DOCX]

Supplementary Material

# Supplementary Figures and Tables

## Supplementary Figures

Figure S1 The retrospective patient recruitment pathway in this study.


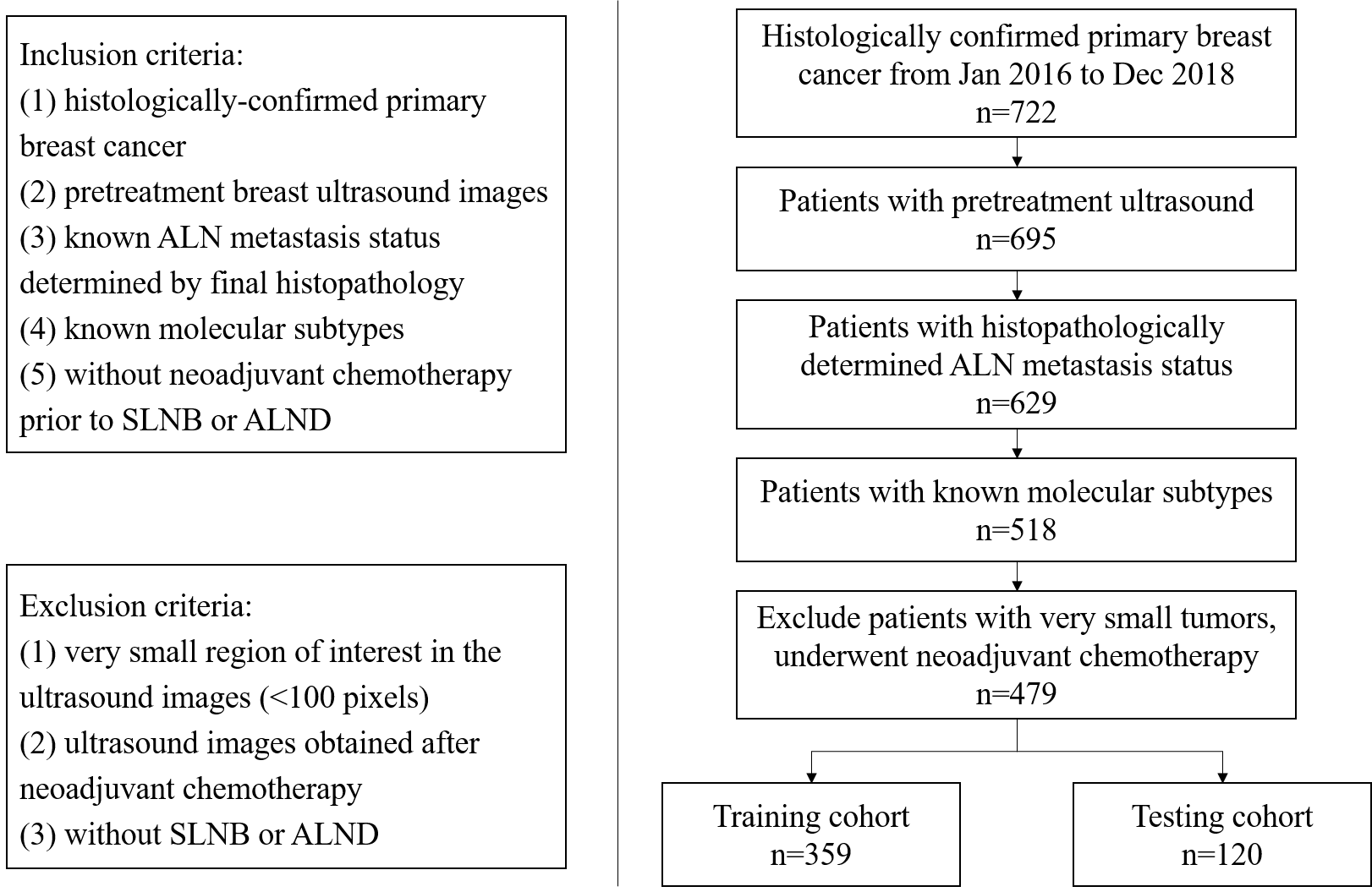


Figure S2 The ROC curves of the three image-only deep CNNs and the three image-only radiomics models in the prospective validation cohort. (A): ROC curves of image-only CNNs in prospective validation cohort. (B): ROC curves of image-only radiomics models in prospective validation cohort.

(A)
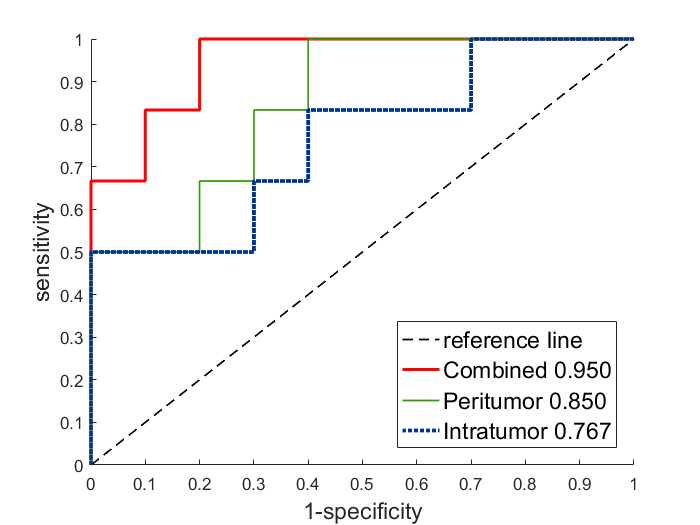
(B)
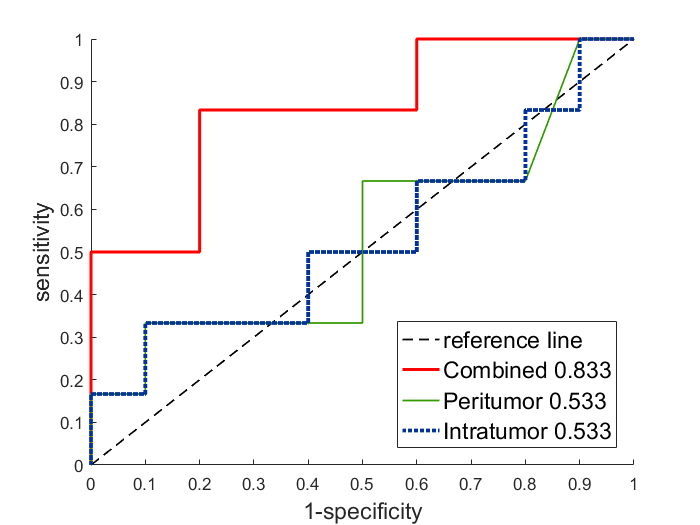


## Supplementary Tables

Table S1 The network configuration used in our study. The network had four Dense Blocks that have 6, 12, 24 and 16 layers, respectively. Each layer consisted of one $1\times1$ convolution and one $3\times3$ convolution. Before the first Dense Block, a $7\times7$ convolution with stride 2 followed by a $3\times3$ max pooling with stride 2 was performed on the $224\times224$input images. A $1\times1$ convolution followed by a $2\times2$ average pooling with stride 2 was used as a transition layer between two adjacent Dense Blocks. At the top of the last Dense Block, a $7\times7$ global average pooling was performed, and a sigmoid node was used to yield the final output. The output size of feature maps at each layer was shown

| **Layers** | **Output Size** | **DenseNet-121** |
| --- | --- | --- |
| Convolution | 112 $\times$ 112 | 7 $\times$ 7 conv, stride 2 |
| Pooling | 56 $\times$ 56 | 3 $\times$ 3 max pool, stride 2 |
| Dense Block (1) | 56 $\times$ 56 | $\left[ \begin{aligned} 1 \times1 conv \\ 3 \times3 conv \end{aligned} \right]$ $\times6$ |
| Transition Layer (1) | 56 $\times$ 56 | 1 $\times$ 1 conv |
|  | 28 $\times$ 28 | 2 $\times$ 2 average pool, stride 2 |
| Dense Block (2) | 28 $\times$ 28 | $\left[ \begin{aligned} 1 \times1 conv \\ 3 \times3 conv \end{aligned} \right]$ $\times12$ |
| Transition Layer (2) | 28 $\times$ 28 | 1 $\times$ 1 conv |
|  | 14 $\times$ 14 | 2 $\times$ 2 average pool, stride 2 |
| Dense Block (3) | 14 $\times$ 14 | $\left[ \begin{aligned} 1 \times1 conv \\ 3 \times3 conv \end{aligned} \right]$ $\times24$ |
| Transition Layer (3) | 14 $\times$ 14 | 1 $\times$ 1 conv |
|  | 7 $\times$ 7 | 2 $\times$ 2 average pool, stride 2 |
| Dense Block (4) | 7 $\times$ 7 | $\left[ \begin{aligned} 1 \times1 conv \\ 3 \times3 conv \end{aligned} \right]$ $\times16$ |
| Classification  Layer | 1 $\times$ 1 | 7 $\times$ 7 global average pool |
|  |  | 1024D fully-connected, sigmoid |

Table S2 A summary of the radiomics features extracted. Informational Measure of Correlation have two calculation methods, respectively, which can be found in the study by Aerts H.J. et al [1].

| **Feature Classes** | | **Feature Names** |
| --- | --- | --- |
| Shape Features | | Volume, Elongation, Surface Area, Max 2D Diameter, Mesh Volume, Major Axis Length, Max 2D Diameter Row, Max 2D Diameter Column, Surface Volume Ratio, Sphericity, Minor Axis Length |
| Intensity Features | | Maximum, Median, Minimum, Mean Energy, Entropy, Variance, Kurtosis, Root Mean Square, Skewness, 10th Percentile, 90th Percentile, Mean Absolute Deviation, Uniformity, Range, Robust Mean Absolute Deviation, Total Energy, Interquartile Range |
| Texture  Features | GLCM  Features | Contrast, Correlation, Autocorrelation, Cluster Tendency, Sum Average, Sum Entropy, Sum Squares, Difference Average, Difference Variance, Difference Entropy, Cluster Prominence, Cluster Shade, Maximum Probability, Inverse Difference Moment, Informational Measure of Correlation 1/2, Inverse Difference Moment Normalized, Inverse Difference Normalized, Inverse Difference, Inverse Variance, Maximal Correlation Coefficient, Joint Average, Joint Energy, Joint Entropy |
|  | GLDM  Features | Dependence Entropy, Dependence Non-Uniformity, Dependence Non-Uniformity Normalized, Dependence Variance, Gray-Level Non-Uniformity, Gray-Level Variance, High Gray-Level Emphasis, Large Dependence Emphasis, Large Dependence High Gray-Level Emphasis, Large Dependence Low Gray-Level Emphasis, Low Gray-Level Emphasis, Small Dependence Emphasis, Small Dependence High Gray-Level Emphasis, Small Dependence Low Gray-Level Emphasis |
|  | GLRLM  Features | Gray-Level Non-uniformity, Gray-Level Non-uniformity Normalized, Gray-Level Variance, High Gray-Level Run Emphasis, Long Run Emphasis, Long Run High Gray-Level Emphasis, Long Run Low Gray-Level Emphasis, Low Gray-Level Run Emphasis, Run Entropy, Run Length Non-Uniformity, Run Length Non-Uniformity Normalized, Run Percentage, Run Variance, Short Run Emphasis, Short Run High Gray-Level Emphasis, Short Run Low Gray-Level Emphasis |
|  | GLSZM  Features | Gray-Level Non-Uniformity, Gray-Level Non-Uniformity Normalized, Gray-Level Non-Uniformity Normalized, High Gray-Level Zone Emphasis, Large Area Emphasis, Large Area High Gray-Level Emphasis, Large Area Low Gray-Level Emphasis, Low Gray-Level Zone Emphasis, Size Zone Non-Uniformity, Size Zone Non-Uniformity Normalized, Small Area Emphasis, Small Area High Gray-Level Emphasis, Small Area Low Gray-Level Emphasis, Zone Entropy, Zone Percentage, Zone Variance |
|  | NGTDM  Features | Coarseness, Contrast, Busyness, Complexity, Strength |

Table S3 A summary of the selected features for building the radiomics models. Impt and pFDR are short for importance measure and false discovery rate (FDR)-adjusted p value. Wilcoxon rank-sum test was performed to assess the univariant association between the selected imaging features and the ALN metastasis status. Multiple hypothesis correction of the p value was performed by using the FDR adjustment based on Benjamini-Hochberg method [2].

(A) The selected features for building the intratumoral radiomics model.

| **No.** | **Selected Features** | **Type** | **Impt** | **p** | **pFDR** |
| --- | --- | --- | --- | --- | --- |
| *f*_1_ | Root Mean Squared | intensity | 9.51 | <.001 | <.001 |
| *f*_2_ | GLSZM.Zone Percentage | texture | 9.08 | <.001 | <.001 |
| *f*_3_ | GLSZM.Size Zone Non-Uniformity | texture | 8.41 | <.001 | <.001 |
| *f*_4_ | GLSZM.Small Area Emphasis | texture | 8.35 | <.001 | <.001 |
| *f*_5_ | GLSZM.Small Area Low Gray-Level Emphasis | texture | 8.11 | <.001 | <.001 |
| *f*_6_ | GLSZM.High Gray-Level Zone Emphasis | texture | 8.11 | <.001 | <.001 |
| *f*_7_ | GLRLM.Gray-Level Variance | texture | 8.08 | <.001 | <.001 |
| *f*_8_ | Median | intensity | 8.03 | <.001 | <.001 |
| *f*_9_ | Mean | intensity | 7.83 | <.001 | <.001 |
| *f*_10_ | GLRLM.Run Entropy | texture | 7.62 | <.001 | <.001 |
| *f*_11_ | GLSZM.Gray-Level Non-Uniformity | texture | 7.56 | <.001 | <.001 |
| *f*_12_ | GLRLM.Gray-Level Non-Uniformity | texture | 7.05 | <.001 | <.001 |
| *f*_13_ | GLSZM.Size Zone Non-Uniformity Normalized | texture | 6.76 | <.001 | <.001 |
| *f*_14_ | Minimum | intensity | 4.3 | <.001 | <.001 |
| *f*_15_ | NGTDM.Coarseness | texture | 2.31 | <.001 | <.001 |

(B) The selected features for building the peritumoral radiomics model.

| **No.** | **Selected Features** | **Type** | **Impt** | **p** | **pFDR** |
| --- | --- | --- | --- | --- | --- |
| *l*_1_ | Elongation | shape | 11.10 | <.001 | <.001 |
| *l*_2_ | GLRLM.Gray-Level Non-Uniformity | texture | 10.57 | <.001 | <.001 |
| *l*_3_ | GLSZM.Size Zone Non-Uniformity | texture | 10.30 | <.001 | <.001 |
| *l*_4_ | Maximum 2D Diameter Row | shape | 9.66 | <.001 | <.001 |
| *l*_5_ | Mesh Volume | shape | 9.30 | <.001 | <.001 |
| *l*_6_ | GLRLM.Run Length Non-Uniformity | texture | 9.28 | <.001 | <.001 |
| *l*_7_ | GLSZM.Zone Entropy | texture | 9.01 | <.001 | <.001 |
| *l*_8_ | Minor Axis Length | shape | 8.89 | <.001 | <.001 |
| *l*_9_ | GLSZM.Gray-Level Non-Uniformity | texture | 8.72 | <.001 | <.001 |
| *l*_10_ | Surface Volume Ratio | shape | 7.89 | <.001 | <.001 |
| *l*_11_ | NGTDM.Coarseness | texture | 3.53 | <.001 | <.001 |

(C) The selected features for building the combined-region radiomics model.

| **No.** | **Selected Features** | **Type** | **Impt** | **p** | **pFDR** |
| --- | --- | --- | --- | --- | --- |
| *g*_1_ | Surface Volume Ratio | shape | 13.51 | <.001 | <.001 |
| *g*_2_ | GLSZM.Zone Entropy | texture | 10.17 | <.001 | <.001 |
| *g*_3_ | GLRLM.Run Length Non-Uniformity | texture | 9.83 | <.001 | <.001 |
| *g*_4_ | Maximum 2D Diameter Row | shape | 9.66 | <.001 | <.001 |
| *g*_5_ | GLSZM.Gray-Level Non-Uniformity | texture | 8.43 | <.001 | <.001 |
| *g*_6_ | GLRLM.Gray-Level Non-Uniformity | texture | 8.40 | <.001 | <.001 |
| *g*_7_ | GLSZM.Size Zone Non-Uniformity | texture | 7.43 | <.001 | <.001 |
| *g*_8_ | Volume | shape | 7.35 | <.001 | <.001 |
| *g*_9_ | Minor Axis Length | shape | 6.93 | <.001 | <.001 |
| *g*_10_ | Surface Area | shape | 6.84 | <.001 | <.001 |
| *g*_11_ | Mesh Volume | shape | 6.81 | <.001 | <.001 |
| *g*_12_ | Sphericity | shape | 6.71 | <.001 | <.001 |
| *g*_13_ | NGTDM.Coarseness | texture | 6.58 | <.001 | <.001 |

**Reference**

[1]Aerts, H.J.,Velazquez, E.R.,Leijenaar, R.T.,Parmar, C.,Grossmann, P.,Carvalho, S. et al. (2014). Decoding tumour phenotype by noninvasive imaging using a quantitative radiomics approach. *Nature communications*. 5**,** 4006. doi: 10.1038/ncomms5006

[2]Benjamini, Y. and Hochberg, Y.J.J.o.t.R.s.s.s.B. (1995). Controlling the false discovery rate: a practical and powerful approach to multiple testing. *Journal of the Royal Statistical Society*. 57**,** 289-300. doi: 10.1111/j.2517-6161.1995.tb02031.x
